# Critical review of the current and future prospects of VEGF-TKIs in the management of squamous cell carcinoma of head and neck

**DOI:** 10.3389/fonc.2023.1310106

**Published:** 2023-12-13

**Authors:** Prashant Puttagunta, Saagar V. Pamulapati, James E. Bates, Jennifer H. Gross, William A. Stokes, Nicole C. Schmitt, Conor Steuer, Yong Teng, Nabil F. Saba

**Affiliations:** ^1^ Medical Education, University of Michigan Medical School, Ann Arbor, MI, United States; ^2^ Internal Medicine Program, Mercyhealth Graduate Medical Education Consortium, Rockford, IL, United States; ^3^ Department of Radiation Oncology, Emory University School of Medicine, Atlanta, GA, United States; ^4^ Department of Otolaryngology – Head and Neck Surgery, Emory University School of Medicine, Atlanta, GA, United States; ^5^ Department of Hematology and Medical Oncology, Emory University School of Medicine, Atlanta, GA, United States

**Keywords:** squamous cell carcinoma of the head and neck, vascular endothelial growth factor, tyrosine kinase inhibitor, immune checkpoint inhibitors, human papillomavirus

## Abstract

As the prognosis for squamous cell carcinoma of the head and neck remains unsatisfactory when compared to other malignancies, novel therapies targeting specific biomarkers are a critical emerging area of great promise. One particular class of drugs that has been developed to impede tumor angiogenesis is vascular endothelial growth factor-tyrosine kinase inhibitors. As current data is primarily limited to preclinical and phase I/II trials, this review summarizes the current and future prospects of these agents in squamous cell carcinoma of the head and neck. In particular, the combination of these agents with immunotherapy is an exciting area that may be a promising option for patients with recurrent or metastatic disease, evidenced in recent trials such as the combination immune checkpoint inhibitors with lenvatinib and cabozantinib. In addition, the use of such combination therapy preoperatively in locally advanced disease is another area of interest.

## 1 Introduction

Prognosis of squamous cell carcinoma of the head and neck (SCCHN) has remained unsatisfactory when compared to other malignancies despite advancements in many treatment modalities (1). Five-year overall survival (OS) estimates of SCCHN range from 30-70% depending on staging and clinicopathologic characteristics (2). Mainstays of treatment have historically been a combination or sole approach of chemotherapy and/or radiation, with surgical approaches primarily restricted to locoregional disease (3). More recently, novel systemic therapies and immunotherapeutics promise change to this treatment paradigm (4) with certain treatments, such as T-cell associated therapeutics, specifically targeting human papillomavirus (HPV) related disease (5).

In addition to HPV-related disease, extensive research is aimed at developing targeted therapies against other biomarkers specific to SCCHN (6); there is also rising interest in the combination of these targeted therapies with immunotherapy (7). Vascular endothelial growth factor (VEGF) is one such pathway that has been recognized as a potential target due to its integral role in angiogenesis and tumor growth, as well as its relationship with immunosuppression (8). One particular class of drugs that has been developed to impede this classical pathway of tumor angiogenesis is VEGF-tyrosine kinase inhibitors (TKIs), which have been implemented in the treatment of solid tumors for over 20 years (9).

This review aims to summarize the literature regarding VEGF-TKIs in solid tumors, specifically in the management of SCCHN, with a broad focus on current and future prospects. With appropriate surveillance and management of on target toxicities, these agents have been shown to be well tolerated (10) and have shown modest benefit in other malignancies, such as renal cell carcinoma (RCC) (11). However, investigation and application to SCCHN particularly is an emerging area primarily restricted to preclinical and phase I/II trials. By reviewing the evidence to date, avenues for future study of VEGF-TKIs in SCCHN can be uncovered.

## 2 VEGF in SCCHN

Angiogenesis is a critical component in the growth and proliferation of many solid tumors and is thought to be essential for rapid tumor growth and metastasis. As tumors outgrow their blood supply and enter a state of relative hypoxia, cancer cells release a cascade of pro-angiogenic factors, including vascular endothelial growth factor (VEGF), fibroblast growth factor (FGF), platelet-derived growth factor (PDGF), and angiopoietins (12). These factors collectively stimulate endothelial cells lining nearby blood vessels to undergo proliferation, migration, and invasion (13). This marks the initiation of angiogenesis, where endothelial cells degrade the basement membrane surrounding blood vessels, enabling them to sprout towards the tumor in a directed manner (14). As the new blood vessels infiltrate the tumor mass, they deliver oxygen and nutrients, allowing the tumor to grow rapidly and potentially gain the ability to metastasize; in SCCHN specifically, increased expression of VEGF is correlated with nodal and distant spread (15, 16). Of the many factors contributing to angiogenesis, VEGF is the most extensively studied, and its action at its various target receptor tyrosine kinase vascular endothelial growth factor receptors (VEGFR) has become a subject of interest as a therapeutic target.

Angiogenesis is a highly regulated phenomenon and contributes to the complexity of the tumor microenvironment, particularly through its relationship with immunosuppression. VEGF is thought to contribute to immunosuppression through several mechanisms, including the upregulation of immune checkpoints such as programmed death ligand-1 (PD-L1) and cytotoxic T-lymphocyte-associated protein 4 (CTLA-4) (17). VEGF expression also influences T-lymphocyte maturation, inhibiting differentiation into CD8+ and CD4+ T cells from hematopoietic stem cells (18). VEGF has also been shown to inhibit natural killer (NK) cell cytotoxicity and dendritic cell (DC) maturation (19, 20). Together, these mechanisms contribute to the relative state of immunosuppression induced by cancer cells through overexpression of immune regulatory cells versus immune effector cells in the tumor microenvironment. The parallels between the mechanisms of angiogenic agents and those of other critical immunosuppressive pathways in various cancers present an argument for the use of angiogenic drugs in conjunction with immuno-oncologic (IO) agents, such as immune checkpoint inhibitors (ICIs) (21).

The role and clinical significance of VEGF and VEGFR expression in SCCHN is thus a topic of ongoing study. Multiple subtypes of VEGF signaling proteins, such as VEGF-A and VEGF-C, have been identified and shown to play specific roles in angiogenesis (22, 23). In a study analyzing 73 tumor samples via immunohistochemistry, the 5-year OS rate of patients with VEGF-C positive and negative oral SCC were found to be significantly different, at 51.7% and 94.2% respectively; VEGF-A, however, was found to be not significantly correlated with survival (24). A separate study utilizing immunohistochemistry in 166 oral dysplasia and tumor samples found increasing expression of VEGF with increasing grades of tumor dysplasia (25). A more recent analysis of 157 tumor samples found VEGF expression to be an independent negative predictor for locoregional control, metastasis-free survival, and OS (26). A meta-analysis of 12 studies found that VEGF overexpression was correlated with an 88% increased risk of death within 2 years, but it was not significantly correlated with nodal and distant spread (27). In contrast, a more recent meta-analysis of several different biomarkers in oral tongue SCC found that increased VEGF-C expression was actually associated with improved survival and VEGF-A and VEGF-C expression together were not significantly prognostic; however, when the single VEGF-C study was excluded from the meta-analysis VEGF-A expression alone demonstrated strong prognostic significance in OS in patients with tongue SCC (28). Overall, the relationship between subtypes of VEGF and their various receptors on pathologic disease progression and patient survival appears to hold some prognostic significance, though to what extent remains unclear and requires further study.

Of note, increased VEGFR2 expression in HPV-related SCCHN has been associated with worse prognosis, and marked differences in expression have been noted in tumor cells and tumor-supporting blood vessels when compared to HPV-negative disease (29). These differences in VEGF expression appear to be partly mediated by HPV-E5/E6 oncoproteins in HPV-related cervical cancer (30), though the mechanism in HPV-related SCCHN is currently less clear. The increasing incidence of HPV-related SCCHN (31) has led to further study into targeted therapies tailored to markers unique to HPV-related disease (5).

There are currently 14 FDA-approved antiangiogenic therapeutics that fall into several different broad classes; ligand directed antibodies, including bevacizumab, ziv-aflibercept, and dalantercept; receptor directed antibodies, of which ramucirumab is the first in class; and tyrosine kinase inhibitors, which are small molecules that exert their effects by binding a vast array of possible angiogenic targets (32, 33). Of these, VEGFR multikinase inhibitors exhibit their effects on VEGFR, PDGFR, and FGFRs, among other members of the VEGFR family, and have demonstrated robust inhibition of angiogenesis in the tumor microenvironment leading to clinical success in many solid tumors (9, 34). However, the application of VEGFR TKIs in the treatment of SCCHN has yet to be fully established.

### 2.1 VEGF-TKI monotherapy in solid tumors

There are currently no FDA-approved antiangiogenesis therapies for SCCHN. Although there was rising interest in using such therapies in the field, bevacizumab in particular, the results of a phase III trial (E1305) of chemotherapy with or without bevacizumab for first line treatment of patients with R/M SCCHN were discouraging. Results demonstrated the addition of bevacizumab improved response rate (RR) and progression free survival (PFS); however, OS was not significantly different between the bevacizumab (12.6 months) group versus with chemotherapy alone (11 months). In addition, there was increased toxicity in the bevacizumab group with 6.7% of patients experiencing grade 3 to 5 adverse bleeding events (35). Subsequent studies have focused on better tolerated agents with a focus on molecular biomarkers as described below.

The use of VEGF-TKIs in other solid organ tumors has been promising and may suggest further study for the use of anti-angiogenic therapies in SCCHN, either as monotherapy or in combination with immune checkpoint inhibitors, two of which are currently approved for use in SCCHN. Given the possible synergistic effects of PD-1 checkpoint inhibitors and anti-angiogenic agents (36), the pairing of ICIs and TKIs is an appealing strategy and has already shown benefit in many tumor types.

Sunitinib is a multikinase inhibitor (MKI) that inhibits VEGFR-1, -2, -3, PDGFR, and protooncogenes c-Kit and RET. It was FDA-approved for metastatic RCC after an international multicenter phase 3 trial demonstrated significant improvements in RR, PFS, and OS (following censoring) vs. IFN-α (37). Further studies elucidated optimal treatment schedules to manage its high toxicity profile (38). It is also approved for treatment of gastrointestinal stromal tumor (GIST) (39).

Sorafenib is an MKI that inhibits VEGFR-1, -2, -3, PDGFR, c-Kit, RET, and protooncogene Raf. It is approved as monotherapy in RCC, hepatocellular carcinoma (HCC), and radioactive iodine (RAI)-refractory differentiated thyroid cancer (40–42). It was initially FDA-approved for use as monotherapy in RCC following the TARGET trial which demonstrated increased PFS and increased likelihood of response or stable disease, though a trend in improvement in OS was initially deemed not statistically significant (40).

Lenvatinib is a MKI of VEGFR, FGFR, PDGFR, RET, and KIT. It was similarly approved for use as monotherapy in patients with RAI-refractory differentiated thyroid cancer after a randomized placebo controlled trial demonstrated improvements in PFS and RR (43). Following a study demonstrating robust improvement in OS, PFS, and overall response rate (ORR) after combination treatment of lenvatinib and everolimus versus either drug alone in advanced or metastatic RCC, it was approved as combination therapy (44).

Axitinib is a second generation TKI specific for VEGFR. Following two stage III trials, axitinib demonstrated improved PFS and ORR versus sorafenib in patients with recurrent metastatic RCC; however, neither trial demonstrated improvements in OS (45, 46). Therefore, the drug is not currently FDA-approved in the first line setting.

Cabozantinib is a recently developed MKI that inhibits VEGFR, as well as AXL, MET, KIT, and RET which are associated with resistance to VEGF inhibitors in RCC (47). Cabozantinib was granted FDA approval after a phase III trial demonstrated improved OS, PFS, and ORR vs everolimus in patients with advanced or metastatic RCC who exhibited disease progression following treatment with other VEGF inhibitors (48). It has also been approved as second line therapy in patients with locally advanced or metastatic RAI-refractory differentiated thyroid cancer who have progressed on first line VEGFR-targeted therapy (49). Given cabozantinib has an extended plasma half-life that leads to drug accumulation during initial dosing, zanzalintinib (XL092) was developed. XL092 retains the same target profile as cabozantinib, as an MKI that inhibits VEGFR, MET, and the TAM kinases (TYRO3, AXL, MER), but has a considerably shorter half-life (50). Currently there is an ongoing multicenter Phase 1b study (NCT05176483) involving dose escalation and cohort expansion of XL092 in combination with immuno-oncology agents. This study is being conducted in patients with unresectable advanced or metastatic solid tumors (51).

### 2.2 Combination of VEGF-TKIs with immunotherapy in solid tumors

The use of VEGF-TKIs and ICIs as combination therapy presents an exciting prospect for the treatment of many different tumor types. Though no combination therapy is currently approved for SCCHN, the combination of ICI and TKI has recently demonstrated benefit in other malignancies. Two initial studies evaluating this combination therapy in RCC were published simultaneously: the Javelin renal 101 and KEYNOTE 426 trials (52, 53). The Javelin renal 101 trial assigned 886 patients with previously untreated advanced RCC to receive avelumab (PD-L1 ICI) plus axitinib (442 patients) or sunitinib alone (444 patients). In patients with PD-L1 positive tumors, PFS was higher in the avelumab plus axitinib group (13.8 months) versus sunitinib alone (7.2 months). This was also true for the overall population with PFS for the avelumab plus axitinib group (13.8 months) versus sunitinib alone (8.4 months). However, OS was not significantly different in the overall population, at 11.6 months and 10.7 months after combination and monotherapy, respectively. Both groups had similar rates of adverse events, with 71.2% and 71.5% of patients experiencing grade 3 or higher events (52).

The KEYNOTE 426 trial treated 432 patients with previously untreated advanced RCC with axitinib plus pembrolizumab (PD-I ICI) and compared them to 429 patients treated with sunitinib alone. After a median follow-up of 12.8 months, OS in the combination therapy group was significantly higher at 89.9% versus 78.3%. Combination therapy also provided improved PFS (15.1 months versus 11.1 months) and ORR (59.3% versus 35.7%). These results were exhibited regardless of PD-L1 receptor status in tumors. Interestingly, though both groups experienced similar rates of adverse events at 75.8% vs 70.6%, only 10.7% of patients undergoing combination therapy discontinued treatment due to these adverse reactions, versus 49.0% of patients taking sunitinib alone (53). These encouraging results led to many more studies of combination TKI ICI therapy.

The CHECKMATE 9ER trial treated 323 patients with previously untreated advanced RCC with nivolumab (PD-1 ICI) plus cabozantinib and compared them to 328 patients treated with sunitinib alone. The combination therapy afforded improved PFS at 12.5 months compared to 8.3 in the combination and monotherapy groups respectively. Probability of survival at 12 months was 85.7% compared to 75.6%, and ORR was 55.7% compared to 27.1%. Again, grade 3 adverse events occurred at similar rates of 75.3 and 70.6% in the respective groups (54).

Finally, running simultaneously to the previous study, the CLEAR trial compared 3 groups among 1069 total patients: lenvatinib plus pembrolizumab (355 patients), lenvatinib plus everolimus (357 patients), and sunitinib alone (357 patients). PFS was 20.8, 14.7, and 9.2 months in the respective groups. When analyzing OS at 24 months, 79.2% of patients in the lenvatinib plus pembrolizumab group, 66.1% of patients in the lenvatinib plus everolimus group, and 70.4% of patients in the sunitinib group survived; the difference between lenvatinib plus pembrolizumab and sunitinib was significantly different, while the difference between lenvatinib plus everolimus and sunitinib was not. Respective ORR were 71%, 53.5%, and 36.1%. Toxicity was slightly higher in this study, most commonly diarrhea, with 82.4%, 83.1%, and 71.8% of patients experiencing one or more grade 3 adverse events in each of the respective groups (55). Overall, these results in RCC were extremely encouraging and redefined the landscape for first-line treatment. As such, further research about the use of VEGF-TKIs and ICIs combinations in SCCHN is an exciting emerging field of study that warrants further attention.

## 3 VEGF-TKIs in SCCHN

Although the use of VEGF-TKIs has been more extensively investigated in other solid tumors, their use in SCCHN is also a growing area of interest. As the landscape of SCCHN rapidly transforms with the use of immunotherapeutic agents and other novel targeted therapies (1), the use of anti-angiogenic agents in conjunction with these emerging therapies or alone is under much scrutiny. A multitude of preclinical and preliminary phase I/II trials exist or are ongoing as detailed below. However, careful selection criteria are warranted as anti-angiogenic agents are known to increase risk of hemorrhages from tumor sites, intracranially, or in microvasculature, as well as risk of venous and arterial thrombosis (56).

Sorafenib was one of the first VEGF-TKIs to be studied in the setting of SCCHN. A phase II trial was conducted in 2007 to assess its efficacy as monotherapy in 26 patients with recurrent or metastatic (R/M) SCCHN who had received first-line chemotherapy for R/M disease. Although the agent exhibited evidence of angiogenesis inhibition through matched immunohistochemistry, clinical responses were limited to a partial response in only 1 patient (3.8%), and the trial was not advanced to further stages. The most common adverse reactions were fatigue (79%), lymphopenia (42%), mucositis/stomatitis (42%), anemia (35%), hand-foot skin reaction (29%), and hypertension (28%). No grade 4 toxicities were observed, and the most common grade 3 toxicities included lymphopenia in 17% and fatigue in 7% of patients (57). A similar phase II trial subsequently investigated sorafenib in 41 patients with R/M SCCHN, although these patients were chemotherapy naive. The response was again minimal, with a single patient experiencing a partial response, yielding an estimated RR of 2%. There were two grade 4 thrombotic reactions during this trial, one asymptomatic pulmonary embolus, and one episode of cerebral ischemia. The most common grade 3 adverse events were hand-foot syndrome, anorexia, nausea, and stomatitis (58).

Three other trials were conducted in 2010 investigating sunitinib (59–61). This agent had previously shown promise in GIST and metastatic renal tumors (37, 39). Similar to sorafenib, however, all three studies showed minimal RR for patients with R/M SCCHN. Some toxicities noted in these trials included worsening of tumor skin ulceration and bleeding complications (61). Additional VEGF-TKIs that have previously been used in small samples of patients with SCCHN, but have not garnered much traction since, include cediranib and motesanib (62, 63).

Despite the limited efficacy of these TKIs, additional agents have continued to be developed and investigated, such as axitinib. Axitinib was first studied in patients with SCCHN in 2015 and administered to 30 patients with unresectable R/M disease. This initial study demonstrated a similarly poor RR compared to prior TKIs at 6.7%; however, results were more promising with an OS of 10.9 months and 76.7% of patients achieving stable disease (64). A follow-up expanded study was conducted by the same researchers on 29 additional patients using a different novel radiographic response criteria (65), which revealed an OS of 9.8 months, 11 patients with partial response, and 1 patient with complete response for an ORR of 42.9%; these results suggested clinical efficacy and the researchers concluded that further study both as monotherapy and in conjunction with immunotherapy is warranted (66). Of note, the combination of axitinib and pembrolizumab has demonstrated promise as a standard of care in advanced clear cell renal carcinoma in phase III trials such as KEYNOTE-426 (67).

Another more recently developed agent is nintendanib, an MKI that targets VEGF, PDGF, and FGF. Historically, it has been used to treat idiopathic pulmonary fibrosis with a more recent focus on applications for non-small cell lung cancer (68, 69). Preclinically, an *in vitro* study investigated the combination of various MKIs with cisplatin in SCCHN cell lines. Nintendanib was noted to exert the greatest synergistic effect with cisplatin chemotherapy (70). Phase I trials in patients with head and neck cancer showed adequate tolerance (71, 72). KCSG-TRIUMPH was a phase II umbrella trial involving patients with platinum-refractory SCCHN, and one arm specifically investigated nintendanib monotherapy in 8 patients with R/M SCCHN. Marked clinical benefit was demonstrated with ORR of 42.9%, OS of 11.1 months, and PFS of 5.6 months (73).

Apatinib is another TKI that has been explored in SCCHN and shown promising efficacy. Preclinical studies of monotherapy and combination with chemotherapy demonstrated efficacy in *in vivo* murine models with inhibition of tumor growth and improved overall survival (74). A phase II pilot study investigated apatinib monotherapy in patients with R/M SCCHN who had failed at least one prior chemotherapy regimen. Treatment was found to have moderate efficacy and tolerable toxicity, with an ORR of 25% (75). Apatinib monotherapy has been investigated in multiple subsequent studies on nasopharyngeal and non-nasopharyngeal SCCHN with encouraging results (76–78). Apatinib has also been shown to be efficacious in ameliorating radiation-induced brain injury amongst patients with head and neck cancer (79). **Table 1** summarizes the aforementioned studies on VEGF-TKI monotherapy in SCCHN.

**Table 1:** VEGF-TKI monotherapy in SCCHN.

Year	Phase	Regimen	Study Population	Number of Patients	Primary Endpoint
2007	II	Sorafenib	R/M SCCHN who had received first-line chemotherapy	26	PR in 1 patient (3.8%); SD in 10 patients (38.5%); PFS 1.8 months; OS 4.2 months
2010	II	Sorafenib	R/M SCCHN who were chemotherapy naive	41	RR of 2%; PFS 4 months; OS 9 months
2010	II	Sunitinib	R/M SCCHN who had received less than or equal to 2 lines of chemotherapy; Cohort 1: patients with ECOG 0-1; Cohort 2: patients with ECOG 2	Cohort 1: 15; Cohort 2: 7	Cohort 1: PR in 1 patient (6.6%), TTP 2 months, OS 4.9 months; Cohort 2: PR in 0 patients, TTP 2.5 months, OS 4.5 months
2010	II	Sunitinib	R/M SCCHN who had received less than or equal to 2 lines of chemotherapy	17	SD in 3 patients (17.6%); TTP 2.3 months; OS 4 months
2010	II	Sunitinib	R/M SCCHN who had PD within 6 months after platinum-based palliative therapy	38	PR in 1 patient (2.6%); SD in 19 patients (50%); PFS 2 months; OS 3.4 months
2010	I	Nintendanib	Advanced solid tumors	61 (of which 2 had head and neck cancer)	No response noted in patients with HNC, well tolerated with majority grade 3 or 4 AE
2023	II	Nintendanib	R/M SCCHN	8	ORR; 42.9%; OS 11.1 months; PFS 5.6 months
2011	II	Pazopanib	R/M NPC who had failed at least 1 line of chemotherapy	33	PR in 2 patients (6.1%); SD in 16 patients (33.3%); OS 10.8 months
2013	II	Famitinib	R/M NPC who have failed greater than or equal to 2 lines of chemotherapy	58	PR in 5 patients (8.6%); SD in 16 patients (27.6%); PFS 3.2 months
2015	II	Axitinib	Unresectable R/M SCCHN who had received less than or equal to 2 lines of chemotherapy	30	PR in 2 patients (6.7%); SD in 23 patients (76.6%); PFS 3.7 months; OS 10.9 months
2021	II	Axitinib	Unresectable R/M SCCHN who had received less than or equal to 2 lines of chemotherapy	29	PR in 11 patients (39.3%); CR in 1 patient (3.6%); ORR 12 patients (42.9%); OS 9.8 months
2017	II	Apatinib	R/M SCCHN who had failed at least 1 line of chemotherapy	10	ORR 25%; DCR 87.5%
2020	II	Apatinib	R/M NPC who had failed at least 2 lines of chemotherapy	51	ORR 31.37% (16/51); OS 16 months; PFS 9 months
2021	II	Apatinib	R/M NPC, median of 3 prior lines of chemotherapy	33	ORR 36.4%; OS 16 months; PFS 5 months
2022	II	Apatinib	R/M SCCHN who had received standard chemotherapy, radiotherapy, and immunotherapy	53	ORR 15.1%; PFS 4.4 months; OS 6.6 months

R/M, Recurrent or Metastatic; SCCHN, Squamous Cell Carcinoma of the Head and Neck; NPC, Nasopharyngeal Carcinoma; PR, Partial Response; SD, Stable Disease; PFS, Progression Free Survival; OS, Overall Survival; RR, Response Rate; ORR, Objective Response Rate; TTP, Time to Progression; AE, Adverse Events; PD, Progressive Disease.

### 3.1 Combination of VEGF-TKIs with chemotherapy or EGFR inhibitors in SCCHN

Results from previous studies on the combination of VEGF-TKIs and other therapeutic agents for patients with SCCHN have been mixed. Apatinib was studied in conjunction with various chemotherapy regimens in a phase II study enrolling patients with R/M head and neck cancer. 47 of the patients enrolled had SCCHN, and patients received axitinib in addition to S-1, capecitabine, nab-paclitaxel, or gemcitabine chemotherapy as tolerated. Preliminary results demonstrated a complete response in 1 patient, a partial response in 3 patients, and an OS of 29.6 months. Final results are forthcoming (80).

Apatinib has also been studied concurrently with S-1 oral chemotherapy as a novel induction therapy in patients with locally advanced SCCHN. 49 patients were enrolled in this single arm phase II trial. Although ultimately survival was minimally altered with a 3-year OS of 64.2%, the ORR was markedly high at 97.4%. Of note, p16 immunohistochemical testing revealed p16 positivity in 14 of 30 oropharyngeal cancer patients tested. Researchers stated one of the limitations of the study was that routine HPV testing had not been practiced in their institution until more recently, and future studies should focus on such testing given HPV has shown to be a prognostic marker (81).

Additional TKIs have been tested concurrently with chemotherapy. One randomized controlled trial investigated docetaxel with or without vandetanib. Although there was a minor trend indicating improved RR of 13% and PFS of 9 weeks in the combination therapy group versus RR of 7% and PFS of 3.2 weeks in the monotherapy group, these results were deemed not clinically meaningful (82). Another randomized controlled trial evaluating the addition of the epidermal growth factor (EGFR) inhibitor cetuximab to sorafenib was conducted as the researchers hypothesized that the two agents may have a synergistic anti-tumor effect; however, the study did not demonstrate significant clinical benefit, with similar RR of 8% and an OS of 9 months and 5.7 months without or with sorafenib respectively (83).

In contrast, a phase II single arm study evaluating the combination of sorafenib with cisplatin and 5-fluorouracil chemotherapy in 54 patients with metastatic nasopharyngeal carcinoma demonstrated clinically efficacious results. RR was 77.8%, with 1 complete response and 41 partial responses. OS was 11.8 months, PFS was 7.2 months, and the investigators ultimately concluded that further randomized controlled trials on the comparison of cisplatin and 5-fluorouracil with or without sorafenib are warranted (84).

Another TKI that has been studied as monotherapy or combined with other agents is pazopanib. Pazopanib is currently approved for the treatment of soft-tissue sarcomas (83) and has shown promise in metastatic RCC (85). Pazopanib was administered in a phase II trial as monotherapy in 33 patients with R/M nasopharyngeal carcinoma. Partial response was demonstrated in 2 patients, stable disease in 16 patients, with an OS of 10.8 months (86). Another phase Ib trial investigated pazopanib combined with cetuximab in 22 patients with R/M SCCHN. 2 patients had complete responses, 9 patients had partial responses, and OS was 9.5 months (87). Pazopanib was tolerated in both trials and showed promising clinical efficacy.

Famitinib is another agent currently under investigation for use in RCC, colorectal cancer, and breast cancer, amongst other malignancies (88–90). One phase II single arm trial of famitinib monotherapy in 58 patients with R/M nasopharyngeal carcinoma showed partial response in 5 patients, stable disease in 16 patients, and PFS of 3.2 months. Researchers concluded that famitinib exhibited substantial clinical benefit and mild-moderate adverse events (91). Phase I trials for famitinib in combination with concurrent chemotherapy also showed moderate clinical efficacy and adequate tolerance in patients with locoregionally advanced nasopharyngeal carcinoma (92). **Table 2** summarizes the aforementioned studies on the combination of VEGF-TKIs with chemotherapy or EGFR inhibitors in SCCHN.

**Table 2:** Combination of VEGF-TKIs with Chemotherapy or EGFR inhibitors in SCCHN.

Year	Phase	Regimen	Study Population	Number of Patients	Primary Endpoint
2013	II	Sorafenib + Cisplatin + 5FU	R/M NPC	54	RR of 77.8%; OS 11.8 months; PFS 7.2 months
2015	II	Sorafenib +/- Cetuximab	R/M SCCHN who had received less than or equal to 1 line of chemotherapy; Arm 1: Cetuximab monotherapy; Arm 2: Combination therapy	Arm 1: 27; Arm 2: 28	ORR 8% for both arms; Arm 1: PFS 3 months, OS 9 months; Arm 2: PFS 3.2 months, OS 5.7 months
2015	I	Nintendanib + Afatinib	Advanced solid tumors	45 (of which 7 had SCCHN)	No response noted in patients with SCCHN, well tolerated with majority grade 3 AE
2019	Ib	Pazopanib + Cetuximab	R/M SCCHN	22	CR in 2 patients (6%); PR in 9 patients (29%); OS 9.5 months
2018	I	Famitinib + Cisplatin	locoregionally advanced NPC	20	PR in 3 patients (15%); SD in 16 patients (80%); 2 patients developed DLT
2013	II	Vandetanib +/- Docetaxel	R/M SCCHN who had received less than or equal to 1 line of platinum-based chemotherapy; Arm 1: Docetaxel monotherapy; Arm 2: Combination therapy	Arm 1: 14, Arm 2: 15	Arm 1: PR in 1 patient (7%), PFS 3.21 weeks, OS, 26.8 weeks; Arm 2: PR in 2 patients (13%), PFS 9 weeks, OS 24.1 weeks
2022	II	Apatinib +/- chemotherapy as tolerated	R/M HNC	73 (of which 47 had SCCHN)	CR in 1 patient; PR in 3 patients; 29 with SD; OS 29.6 months
2023	II	Apatinib + S-1	locally advanced SCCHN	38	ORR 97.4%; 3-year OS 64.2%; 3-year PFS 57.1%
2023	I	Cabozantinib + Cetuximab	R/M SCCHN	22	PR in 4 patients (18.2%); SD in 16 patients (75%); OS 8.1 months; PFS 3.4 months

5FU, Fluorouracil; R/M, Recurrent or Metastatic; SCCHN, Squamous Cell Carcinoma of the Head and Neck; NPC, Nasopharyngeal Carcinoma; HNC, Head and Neck Cancer; PR, Partial Response; SD, Stable Disease; PFS, Progression Free Survival; OS, Overall Survival; RR, Response Rate; ORR, Objective Response Rate; AE, Adverse Events; CR, Complete Response; DLT, Dose Limiting Toxicity.

### 3.2 Combination of VEGF-TKIs with immunotherapy in SCCHN

With VEGF-TKIs exhibiting modest benefit in SCCHN to date, either as monotherapy or in combination with chemotherapy or EGFR inhibitors, a more enticing area of interest is their use in combination with immunotherapy. The synergetic effects of combining VEGF-TKIs and ICIs has been noted in multiple clinical head and neck cancer studies (36, 93, 94). Besides the role of combination therapy as an initial approach, studies have suggested the addition of VEGF-TKIs may also play a role in clinical situations requiring immunotherapy retreatment. A recent trial of famitinib and camrelizumab (PD-1 ICI) was conducted in 18 patients with R/M nasopharyngeal carcinoma who had already progressed on one line of platinum-based chemotherapy and anti-PD-L1 immunotherapy. Results were encouraging, and ORR was 33.3% with a PFS of 7.2 months. These results supported the hypothesis that the addition of concurrent antiangiogenics during immunotherapy retreatment may increase efficacy (93). The same group had previously shown a similar synergistic effect when investigating the combination of apatinib and camrelizumab in patients with R/M nasopharyngeal carcinoma (94).

Concurrent use of lenvatinib and pembrolizumab has also been studied in two trials (95, 96). The phase Ib/II KEYNOTE-146 study investigated this combined regimen in 137 patients with various malignancies, including 22 patients with metastatic SCCHN. 8 patients achieved a response (36%) (96). The subsequent phase II study in 14 patients with heavily pretreated R/M SCCHN treated with lenvatinib and pembrolizumab demonstrated a similar RR of 28.6%, with OS of 6.2 months and PFS of 4.6 months (95). Additional investigations regarding lenvatinib use in SCCHN are ongoing. LEAP-009 (NCT04428151) is a randomized control trial investigating lenvatinib +/- pembrolizumab in comparison to standard-of-care chemotherapy in SCCHN (97). LEAP-010 (NCT04199104) was another randomized control trial comparing pembrolizumab monotherapy to its combination with lenvatinib as first line therapy for PD-L1 positive R/M SCCHN patients, though a recent press release indicated that the trial was closed based on lack of observed significant difference in the primary endpoint of overall survival. No further details were available at the time of this publication (98).

Regorafenib is currently approved in the United States for the treatment of HCC, GIST, and colorectal cancer, among others (99, 100). Addition of regorafenib has been shown to improve the efficacy of anti-PD-L1 immunotherapy in oral squamous cell carcinoma animal models (101). This agent has also shown some clinical efficacy in SCCHN patient derived xenografts (102). One ongoing trial (NCT04704154) is investigating the combination of nivolumab immunotherapy and regorafenib in patients with various solid tumors, including one cohort of those with R/M SCCHN who are immunotherapy naive (103).

Another TKI that has shown recent promise is cabozantinib. Overexpression of c-MET and AXL tyrosine kinase receptors has been demonstrated in cisplatin- and radiotherapy-resistant SCCHN, and cabozantinib targets both receptors. In preclinical trials, administration of cabozantinib was efficacious in mice, zebrafish, and specimens of human SCCHN (104). A follow-up phase I study investigated concurrent cabozantinib and cetuximab therapy in 22 patients with R/M SCCHN. 4 patients exhibited a partial response, of which 2 had prior cetuximab resistance. Stable disease was noted in 75% of the population, with an OS of 8.1 months and PFS of 3.4 months (105). Of note, a recent phase II single arm trial of cabozantinib and pembrolizumab was conducted in 36 R/M SCCHN patients. Partial response was demonstrated in 17 patients, stable disease in 13 patients, with an OS of 22.3 months and PFS of 14.6 months (106). These results further reinforced the enticing prospect of combining VEGF-TKIs and immunotherapy. A more recently promising agent is zanzalintinib (XL092). STELLAR-305 is a randomized, double-blind, phase 2/3 study that will evaluate the efficacy and safety of zanzalintinib plus pembrolizumab vs pembrolizumab and placebo in patients with previously untreated, PD-L1-positive, recurrent or metastatic SCCHN. **Table 3** summarizes the aforementioned studies on the combination of VEGF-TKI with immunotherapy in SCCHN.

**Table 3:** Combination of VEGF-TKIs with immunotherapy in SCCHN.

Year	Phase	Regimen	Study Population	Number of Patients	Primary Endpoint
2023	II	Famitinib + Camrelizumab	R/M NPC who had received at least one line of platinum-based chemotherapy and anti-PD-L1 immunotherapy	18	ORR 33.3%; PFS 7.2 months
2023	II	Apatinib + Camrelizumab	R/M NPC who had received at least 1 line of chemotherapy	58	ORR 65.5%; DCR 86.2%; PFS 10.4 months
2020	Ib/II	Lenvatinib + Pembrolizumab	R/M SCCHN who had received less than or equal to 1 line of chemotherapy	22	RR of 8/22 patients (36%)
2021	II	Lenvatinib + Pembrolizumab	R/M SCCHN who had received less than or equal to 2 lines of chemotherapy	14	RR of 28.6%; OS 6.2 months; PFS 4.6 months
2023	II	Cabozantinib + Pembrolizumab	R/M SCCHN	36	PR in 17 patients (51.5%); SD in 13 patients (39.4%); OS 22.3 months; PFS 14.6 months

R/M, Recurrent or Metastatic; SCCHN, Squamous Cell Carcinoma of the Head and Neck; NPC, Nasopharyngeal Carcinoma; PR, Partial Response; SD, Stable Disease; PFS, Progression Free Survival; OS, Overall Survival; RR, Response Rate; ORR, Objective Response Rate; DCR, Disease Control Rate.

### 3.3 Data in the preoperative setting

Studies evaluating VEGF-TKIs in the preoperative setting for SCCHN are limited. In contrast, there has already been extensive research into the use of EGFR inhibitors, such as cetuximab, and single agent or double agent ICI preoperatively (107–110). Although the majority of studies detailed above are focused on R/M disease, one study to be highlighted that was previously described is the recent 2023 study on apatinib and S-1 in patients with locally advanced SCCHN (81). Typically, the current treatment guidelines for locally advanced disease recommend concurrent chemoradiation, although newer approaches such as induction chemotherapy prior to (chemo)radiation have complicated the picture (111–113). Regardless, lingering concerns regarding the known toxicities of concurrent chemoradiation after induction chemotherapy have engendered further research into newer investigational approaches using ICIs or EGFR inhibitors (114–117).

The use of induction chemotherapy to convert unresectable or borderline resectable disease to definitively resectable disease is also controversial, as results are mixed as to whether this strategy improves survival (118, 119). That being said, the use of apatinib and S-1 allowed for three patients with unresectable SCCHN to undergo curative-intent surgery post therapy (81). The use of VEGF-TKIs preoperatively and for downstaging prior to procedures has been investigated and shown promise in a wide variety of other malignancies (120–123). Cabozantinib in particular has shown promise in the preoperative setting in RCC (124). In addition, the combination of VEGF-TKIs and immunotherapy in the preoperative setting is also a growing area of study (125–127). Already, there exists precedent for using a combinatorial preoperative approach of VEGF-TKIs with ICIs in other malignancies, such as in RCC and HCC (125–127). The toxicity profile of such an approach in SCCHN may perhaps be desirable when compared to standard concurrent chemoradiation (128). Our knowledge is currently limited in this area, given that the use of VEGF-TKIs in the preoperative setting for SCCHN was only explicitly discussed in the one above mentioned clinical study.

## 4 Discussion

As outlined in this review, VEGF-TKIs are diverse in their targets, toxicities, and effectiveness. This makes it difficult to select which TKIs to study, particularly in the setting of SCCHN with early and non-definitive data in the field. The use of VEGF-TKIs in R/M RCC has demonstrated vastly varying effectiveness and toxicities even among agents with multiple overlapping targets; for example, axitinib and sorafenib were found to have similar effectiveness, although axitinib has a much more manageable toxicity profile (46). Combinations with ICIs further complicate the matter, with improved effectiveness at the cost of worsening toxicity (52). Additionally, agents such as sunitinib that demonstrate marked effectiveness in other malignancies such as RCC seem to have little to no effect in SCCHN but with worse toxicity (61). As such, much intentionality is required when choosing agents to study, whether selecting a new agent such as cabozantinib or an older agent with unremarkable results in other malignancies.

The toxicity profiles of VEGF-TKIs are diverse and require careful consideration. Intended selective inhibition of angiogenic tyrosine kinases, in an excessive manner, can lead to on target toxicities, such as skin reactions, hypertension, hypothyroidism and proteinuria (129). However, as VEGF-TKIs have been shown to have promiscuous activity (130), unintended inhibition of non-angiogenic tyrosine kinases can lead to off target toxicities. Other cardiotoxic and hepatotoxic side effects, particularly rare pancreatic enzyme elevations, fatal hepatotoxicity, hypoglycemia, and corrected QT prolongation, have been hypothesized to be partly or fully secondary to these off target effects (131). Additional cardiotoxic side effects, such as left ventricular systolic dysfunction, heart failure, and arterial thromboembolism, have been studied in more detail and are thought to be a result of both on target and off target inhibition (132, 133). The most common side effects of VEGF-TKIs include fatigue, diarrhea, skin toxicity, bleeding and vascular complications, and a variety of laboratory abnormalities (134, 135). These vary greatly among TKIs; for example, sorafenib and regorafinib were found to be the worst offenders for increasing risk for hemorrhagic events at odds ratios of 3.31 and 2.92 respectively (136). With regards to hypertension, a meta-analysis across multiple malignancies found axitinib and sunitinib to increase systolic and diastolic blood pressure the most during treatment, while cabozantinib and sorafenib had the smallest effect (10). With many anti-angiogenic agents to choose from, and even more immuno-oncologic agents to pair them with, studying these agents in a novel setting provides a challenging yet unique opportunity to tailor therapy for SCCHN to a very high degree to individual settings and patients.

It would be of relevance to decipher the characteristics of each of the VEGF-TKIs, namely their immunomodulatory effects and possible benefits or advantages of certain agents over others, especially in the setting of combinatorial effects with immunotherapeutic agents such as PD-1 inhibitors. Along those lines, it is of interest that lenvatinib, despite its clinical activity in combination with pembrolizumab, did not lead to an improvement in OS in a phase III randomized trial (98). Cabozantinib does have other immunomodulatory targets besides VEGF (TAM, MER, cMET) and could possibly result in a stronger combinatorial effect with PD-1 inhibitors in a disease such as SCCHN (47). Despite the encouraging results of cabozantinib in combination with pembrolizumab in recurrent metastatic SCCHN (106), this possible advantage to cabozantinib remains to be speculative at this stage and will require confirmation in a randomized clinical trial. Furthermore, pre-clinical studies using combination of these agents with PD-1 inhibitors in cell lines or syngeneic transplantable mouse oral cancer models (MOC 1 and 2) may help shed some light as to the advantages of one versus the other agent.

Currently, evaluation of patient biomarker expression is not routinely performed prior to treatment of SCCHN with VEGF-TKIs. Precedent for biomarker selection in this patient population exists for treatment with checkpoint inhibitors such as pembrolizumab, where patients whose tumors express PD-L1 have been shown to clinically benefit from treatment (137–139). However, these results are limited by small sample sizes, inconsistent assays, and subjective scoring systems (140). Further, no biomarkers beyond PD-L1 combined positive score (CPS), established in the first line setting based on KEYNOTE 048, have resulted in a practice-changing recommendation. However, two more recent analyses of these pivotal early studies continue to demonstrate significant associations between PD-L1 expression, tumor mutational burden (TMB), and response to ICI treatment; both studies also demonstrate a consistent effect regardless of tumor HPV status (141, 142). However, in our recent study, TMB did not appear to correlate with likelihood of response to treatment with pembrolizumab and cabozantinib (106). As such, with respect to anti-angiogenic treatment, it remains unclear if biomarker selection is worthwhile, considering all tumors rely on angiogenesis to some extent for growth and metastasis. The relationship between variation in treatment response following VEGF-TKIs and respective biomarker expression is still largely unknown and has not been typically evaluated prior to treatment in the various clinical studies outlined above. Given the known correlations between levels of VEGF expression and tumor dysplasia (25) and lowered rates of survival (24), testing patients’ tumors for VEGF expression prior to treatment may be a plausible method to estimate possible effectiveness of anti-angiogenic treatment. However, this would require a viable assay for measuring expression of VEGF and its various receptors, which could prove difficult (143, 144). In addition, most VEGF-TKIs have multiple and varied targets, for which the relationship between expression and treatment effectiveness is largely unexplored. Finally, given the known relationship between angiogenesis and immunosuppression in the tumor microenvironment (21, 145), the potential effects of anti-angiogenic agents may be underestimated without also accounting for tumor expression of related biomarkers such as PD-1/PD-L1 and the effects VEGF-TKIs may have on these pathways, or accounting for combination therapy. As such, biomarker selection may be redundant or unnecessary in the setting of multi-target anti-angiogenic therapy with or without combination ICI therapy, though more understanding is still required regarding the relationship between biomarkers and VEGF inhibitors.

In addition to current uncertainty regarding VEGF-TKI use in SCCHN, ambiguity regarding their use in HPV-related disease further complicates the picture. Just as angiogenesis plays a major role in influencing the tumor microenvironment, HPV-positive disease has also been shown to have an entirely distinct microenvironment when compared to HPV-negative SCCHN (146, 147). It is unclear how this may impact response to VEGF-TKI therapy, given that HPV-related disease engenders marked differences in antigen presentation based on viral oncoproteins and increased activation of immune infiltrates (148, 149). Despite this, there is no clear evidence that these combinatorial approaches are less effective in one versus the other disease, making them more attractive. The added lack of a treatment related biomarker for patient selection may also be an advantage as it adds to the ease and simplicity of using these combinatorial approaches.

As we look towards the future of care for SCCHN, studies on VEGF-TKIs are encouraging for broadening use case scenarios in a disease that still has limited therapeutic options. Recent studies on the combination of VEGF-TKIs and immunotherapy indicate that such an approach may be promising; in addition to the clinical benefit of both classes of agents, the lack of additive side effects with ICIs, and the broad applicability given no required biomarker or patient selection, all point to a high potential for a promising strategy in treating SCCHN and potentially other SCCs. As cancer care and research increasingly move towards targeted therapies and personalized care, VEGF-TKIs in combination with immune checkpoint inhibitors are an important class of therapeutics to focus on in SCCHN and other diseases.

## Author contributions

PP: Conceptualization, Writing – original draft, Writing – review & editing. SP: Conceptualization, Writing – original draft, Writing – review & editing. JB: Writing – review & editing. JG: Writing – review & editing. WS: Writing – review & editing. NS: Writing – review & editing. CS: Writing – review & editing. YT: Writing – review & editing. NS: Conceptualization, Supervision, Writing – review & editing.
